# 100-m resolution Age-Stratified Population Estimation from the 2020 China Census by Township (ASPECT)

**DOI:** 10.1038/s41597-025-05401-1

**Published:** 2025-06-21

**Authors:** Yang Ju, Ying Liang, Jinyu Kong, Xuelu Wang, Shicheng Wen, Huiyan Shang, Xize Wang

**Affiliations:** 1 School of Geography and Ocean Science, Nanjing University, Nanjing, China; 2 School of Architecture and Urban Planning, Nanjing University, Nanjing, China; 3 Department of Architecture, National University of Singapore, Singapore, Singapore; 4 Department of Real Estate, National University of Singapore, Singapore, Singapore

**Keywords:** Geography, Society, Environmental social sciences

## Abstract

Gridded population datasets are instrumental for modeling the interactions between human and the environment at fine spatial scales. Many of these datasets are downscaled from source data of aggregated population counts by census units. Here, we introduce an Age-Stratified Population Estimation from the 2020 China Census by Township (ASPECT), estimating total population and population by age groups (0–14, 15–59, 60–64, ≥65 years old) at 100 m spatial resolution as of year 2020. ASPECT uses the updated source data from the most recent Census of year 2020, incorporating population counts and age structures from nearly all townships (n = 40,718) – the finest spatial unit for which the 2020 Census data are publicly available. Therefore, ASPECT likely provides improved accuracy in gridded population estimation when compared with datasets based on county-level data such as WorldPop. Furthermore, ASPECT presents greater spatial variations in the estimated population age structure than those from other existing datasets. These advantages of ASPECT allow for more accurate estimations on population exposure to environmental hazards and access to public services.

## Background & Summary

Spatially explicit datasets on population distribution are instrumental for understanding human interactions with the environment, facilitating downstream studies on environmental health^1^, disaster management^2^, urban ecology^3^, and racial segregations^4^. Furthermore, since children and older adults are often considered as vulnerable populations, spatially explicit population datasets with age structures are often of greater value in research applications^5^.

Prior studies often utilize population data organized by census units to map population distribution. For example, one study finds that US census block groups with a higher proportion of older adults are more exposed to flooding risks from sea level rise^5^. However, census units vary in size and may contain a large portion of uninhabited areas, and they assume a uniform population distribution within a given unit (which is rarely realistic). These limitations post challenges for effectively modeling population spatial distribution and comparisons between different geographic locations. Therefore, some studies take a further step to use gridded population datasets that refines population distribution within the census unit. For example, Alegana *et al*. estimated the proportion of population under five years old in 1 × 1 km grid cells in Nigeria^6^. They found that accounting for the fine-scale spatial variations in age structure, rather than assuming it being uniform in a census unit, can lead to significant differences in the estimated health metrics. These examples highlight the value of having spatially explicit population datasets at fine spatial resolutions.

**Fig. 1:**
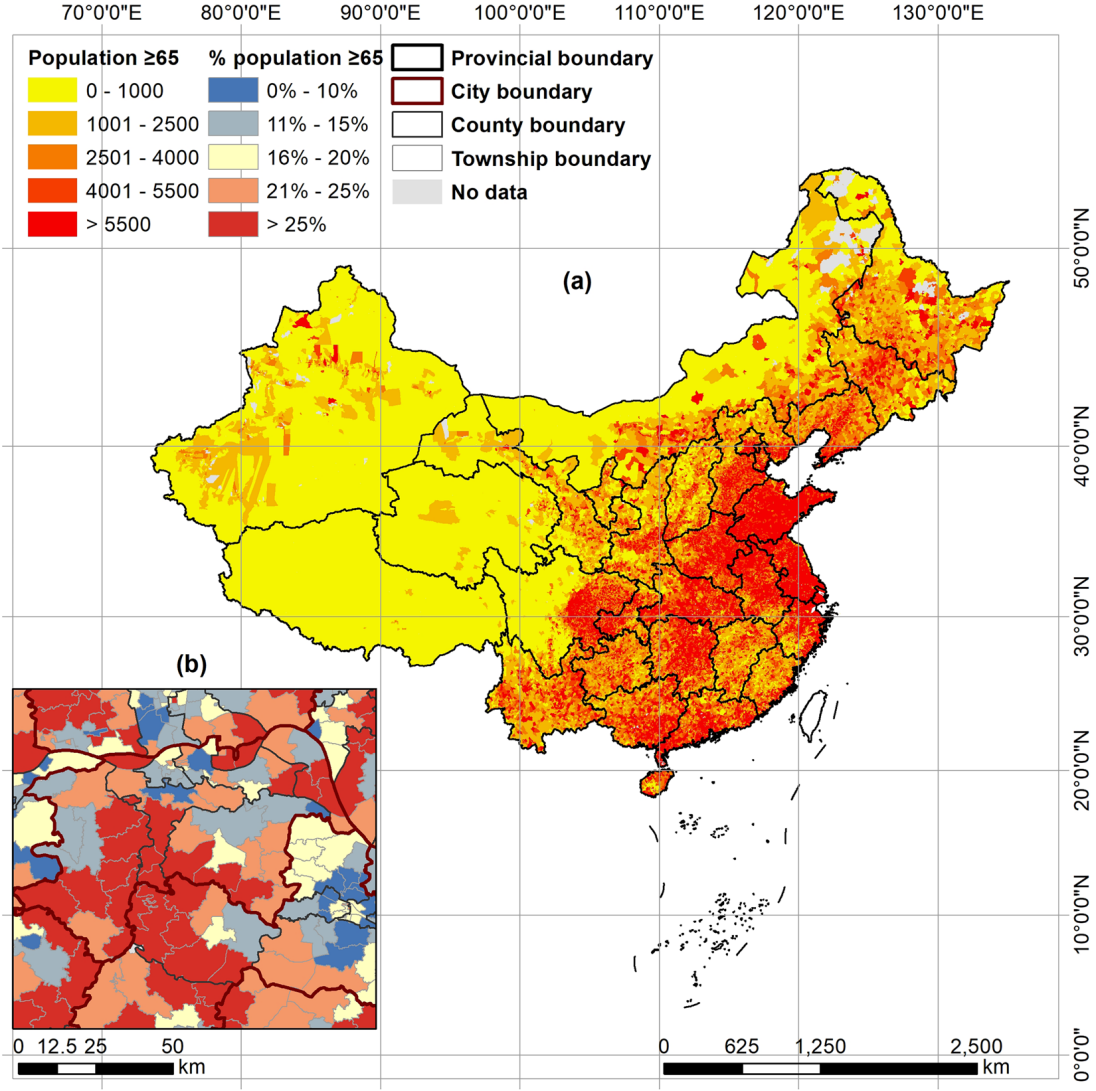
Study area, mainland China (**a**), and boundaries of city, county (district), and township (**b**). Population ≥65 and the proportion of this population are also illustrated at the township level in (**a**) and (**b**), respectively.

While fine-resolution population datasets can be more accessible in some developed economies such as the US and Canada, data accessibility in developing countries like China is less ideal. As a comparison, the US Census and American Community Survey provide data organized by hierarchical spatial units of block, block group, tract, county, and state. The finest spatial unit, block, often corresponds to a street block in urbanized areas and provides information such as total population, racial and ethnical composition, and age structure. The China Census data are organized in a similar hierarchical structure but at coarser spatial resolutions, with the finest spatial unit being the township (Fig. 1). Another challenge for using the township-level China Census data is the lack of timely updated geospatial boundaries for the townships, which makes georeferencing the data difficult. To our best knowledge, the most recent Chinese township boundaries available to the public reflects the conditions of 2019. However, due to township boundary modifications and name changes, there are mismatches between the 2019 township boundary and the 2020 Census data. More specifically, according the Ministry of Civil Affairs, 768 townships in China experienced boundary changes, mostly in forms of merging and splitting into new townships^7^. Updating these boundary changes is a time-consuming but necessary step to properly georeference the 2020 Census data.

Researchers have also resorted to gridded population datasets such as WorldPop and LandScan to obtain population distribution. Briefly, these datasets employ dasymetric mapping methods to disaggregate population counts from census units (source zones) to finer-resolution grid cells (target zones)^8^. Ancillary data that correlate with population distribution and have fine spatial resolutions are used in this disaggregation. These data typically measure the distribution of road, land cover, built structures, topography, elevation, nighttime light intensity, and water bodies, with spatial resolutions as fine as sub-100 meters. Regression models are used to model the relationship between population counts and the ancillary data variables at the census unit level. The resulting models are then applied to the ancillary data in gridded format to predict population count per grid cell.

In China, existing gridded population datasets are often disaggregated from outdated source population data. Furthermore, county (referred to as district in urban areas) is often used as the source zone, whose coarse spatial resolution may compromise the quality of the gridded population dataset. For example, the widely used WorldPop (version 2000–2020) provides total population and population by age groups and sex at 100-m spatial resolution, annually between 2000 and 2020. Specifically, WorldPop (version 2000–2020) uses the 2010 China Census data at township level to estimate gridded total population, and it uses the Census data at county level to estimate gridded population by age groups as of 2010^9,10^. Prefecture-level (one level coarser than county – and often referred as “city-level”) population growth rate is applied to project population for the subsequent years^10,11^. This coarse resolution of prefecture may fail to capture finer-scale population changes at county and township levels. A recent and ongoing update of WorldPop (version 2015–2030, release R2024B V1) aims to incorporate county-level data from the most recent 2020 Census^12^. Although it still relies on county-level population data, this update could help address some inaccuracies in total population and age group estimates in WorldPop (2000–2020) caused by outdated census data and prefecture-level growth rates^12^. A few other datasets on China have also used county as the source zone to produce their gridded population estimates^13,14^. Although more time consuming to incorporate, township-level data can better reflect spatial variations of population in China. One recent dataset, PopSE, represents the first effort to leverage the 2020 Census township-level population data to estimate grid-level population density^15^. However, grid-level population density by specific age groups, which is also available from the 2020 Census and important for analyzing exposures for vulnerable groups, is also needed.

To improve the source data resolution and timeliness, we developed a 100-m resolution Age-Stratified Population Estimation from the 2020 China Census by Township (ASPECT). ASPECT intends to cover mainland China, and the dataset has two main advantages. First, we use population data from 40,718 townships from the most recent 2020 Census to train our random forest model for dasymetric mapping; second, in addition to total population, ASPECT also estimates population by age groups (0–14, 15–59, 60–64, ≥ 65 years old). These additional pieces of information allow for age-group-specific estimates on exposures to environmental hazards and access to environmental goods and public services.

## Methods

### Overview

**Fig. 2:**
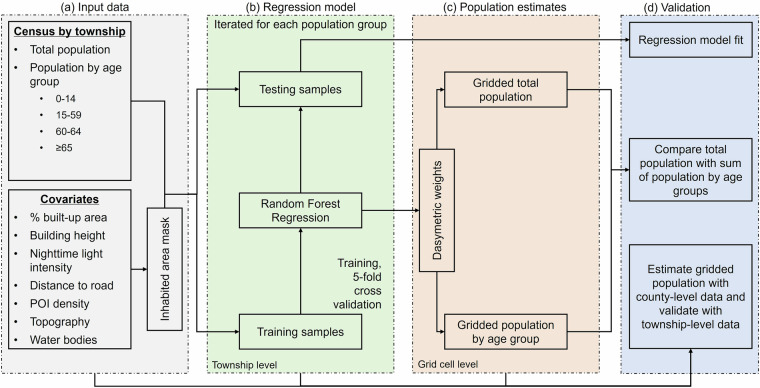
Overall workflow of ASPECT.

We used a dasymetric mapping approach, similar to the ones by previous studies, to generate ASPECT^11,15^. Specifically, we collected a rich set of covariates that predict population distribution (Fig. 2a). Data on these covariates are in forms of 100 m resolution grid cells, which were then aggregated by townships. Using the township level data, we trained a random forest model to regress population counts on the covariates (Fig. 2b). We then applied this model to the gridded covariates to generate a population weighting layer, using which we distributed township-level population to each grid cell (Fig. 2c). Notably, we trained separate random forest regression models and conducted subsequent population weighting procedures separately for each population group (i.e., total population, age group 0–14, age group 15–59, age group 60–64, and age group 65 and older).

We performed three sets of validations to assess the quality of ASPECT (Fig. 2d). First, we evaluated the goodness-of-fit of the township-level regression model. For each grid cell, we then checked whether the estimated total population equaled the sum of estimated population by age groups, as they were estimated from sperate dasymetric mapping processes. Lastly, we performed the dasymetric mapping using data at the county level, which is one administrative level coarser than township. We aggregated gridded population estimates from this county-level mapping by townships and compared with actual township-level values. The comparison helps to justify the validity of the dasymetric mapping, with the assumptions that estimations should be more accurate when the township-level data are used. A comparison with existing datasets, including WorldPop version 2000–2020 (the most recent final release), WorldPop version 2015–2030 (the ongoing update, release R2024B V1), and PopSE by Chen *et al*.^15^ is also provided.

### Population data

We collected township-level population from the Tabulation on 2020 China Population Census by Township^16^. This 2020 Census dataset measures residential population as of Nov 1, 2020, providing information on total population, and population by age groups of 0–14, 15–59, ≥60, and ≥65 years old. The name of each township, along with the names of its higher administrative levels—county, city, and province—are also provided, which we used to geocode the point location (i.e., the longitude and latitude coordinates in the WGS84 system) of each township with the Baidu Map geocoding API (https://lbsyun.baidu.com/).

**Fig. 3:**
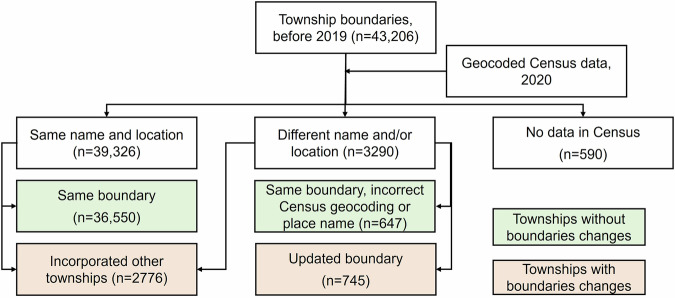
Workflow of updating township boundaries (as of 2019) to match with Census data (2020).

We further collected the administrative boundaries of the townships and linked them with the 2020 Census dataset. We obtained a version of township boundaries that were released by the National Platform for Common Geospatial Information Services (https://www.tianditu.gov.cn/). Based on our observations, these boundaries are likely to reflect the conditions as of 2019. However, according to the Ministry of Civil Affairs, China, boundary changes take place in certain townships between the years^7^. Therefore, we manually updated the 2019 township boundaries to match with the 2020 Census data. Specifically, we first identified 39,326 townships with their names and locations matched between the 2020 Census (with point locations) and the 2019 township boundaries (i.e. the same name and the point falls inside the township boundaries), which we considered as having no boundaries changes (n = 36,550), or having incorporated other townships (n = 2,776). We edited the remaining, mismatched township boundaries by cross-referencing them with government announcements (e.g., Chengdu Civil Affairs Bureau^17^), publicly available map documents (e.g., Chongzhou Municipal Government^18^), and other boundary datasets such as OpenStreetMap^19^. The edits involved updating township names, redrawing boundaries, and fixing incorrect Census geocoding, ensuring the updated township boundaries align with the geographical representation of the census data. Importantly, these edits were limited to the administrative boundaries and Census geocoding, without altering population counts in the Census data. After these edits, we obtained 1,392 additional townships with matching 2020 Census data. Out of these, 745 townships had their administrative boundaries revised, while 647 maintained their original boundaries. Together, townships with boundary changes covered an area of 502,418 km^2^ (5.16% of the total area of mainland China). 590 townships in the 2019 township boundary dataset failed to match with any 2020 Census data (Fig. 3). These townships typically have keywords of state-run farms, forest plantations, and industrial parks in their names, which may have little residential population. We treated these townships as missing data from the 2020 Census. An illustration of the updated township boundaries with 2020 Census data is provided in Fig. 1a, indicating a nearly complete population data coverage of the study area.

### Covariates predicting population distribution

As the next step, we collected data on covariates that correlate with population distribution. These covariates include % built-up area, building height, nighttime light intensity, distance to road, density of Point of Interest (POI), topography, and water bodies. Definitions and data sources of these covariates are in Table 1. We calculated the covariate values for each grid cell and then aggregated them into township-level. These aggregated covariates served as inputs for the subsequent analysis.

**Table 1:** Covariates used to predict population distribution and their definition and data sources.

Covariate	Definition	Data sources and URLs
% built-up area	% built-up area per 100 by 100 m grid cell as of 2021	ESA WorldCover 10 m v200^22^ (https://worldcover2021.esa.int)
Building height	Average building height per 100 by 100 grid cell as of 2020	CNBH-10m^23^ (https://zenodo.org/records/7827315)
Nighttime light intensity	Annual mean nighttime light intensity as of 2020	VIIRS Nighttime Day/Night Annual Band Composites V2.1^24^ (retrieved via Google Earth Engine, https://earthengine.google.com/)
Distance to road	Distance to the nearest road (primary, secondary, and tertiary roads combined), with data collected in 2022	OpenStreetMap^19^ (https://download.geofabrik.de/)
Point-of-Interest (POI) density	Kernel density of POIs	AMap POI as of 2020 (https://lbs.amap.com/)
Topography	Land elevation and slope	NASA SRTM Digital Elevation 30m^25^ (retrieved via Google Earth Engine, https://earthengine.google.com/)
Water bodies	Surface water bodies as of 2020	JRC Yearly Water Classification History, v1.4^26^ (retrieved via Google Earth Engine, https://earthengine.google.com/)

Similar to Chen *et al*.^15^, we used the gridded covariates to generate a mask of inhabited zones to improve the quality of gridded population estimates. An inhabited zone should have % built-up area or building height greater than zero, and it should not be covered by any water bodies. We performed dasymetric mapping only within the inhabited zones, and we treated population estimates outside these zones as zero.

### Dasymetric mapping to estimate gridded population

We started by training a series of random forest models to measure the relationship between population density and the covariates at the township level. The model was trained separately for total population and population by age groups (0–14, 15–59, ≥60, and ≥65 years old), therefore allowing differential covariate effects in population density predictions. Population density was calculated by dividing the township-level population by the area of inhabited zones. We used random forest model following prior literature^11,15^, allowing us to flexibly model nonlinear relationships between population density and the covariates. Notably, we did not log transform population density, as pervious literature has done^11,15^. A log transformation will compress the regression error for extremely densely populated townships, which could be less straightforward how well the covariates predict township-level population density (i.e., our first validation).

We tuned the random forest model using parameters of the number of trees and the maximum depth of each tree. Specifically, we used the townships within the middle 99% range of population density distribution as the candidate to derive training and validation samples. The training sample contains 85% of the candidate townships, and the testing sample contains the remaining 15%. We then tuned the model using the grid search cross validation method, which iteratively trained the random forest model with every unique combination of the tuning parameters’ candidate values. The candidate values for the number of trees included 5, 10, 20, 40, 60, 80, 100, 150, 200, 400, 600, 800, and 1000, and the candidate values for the maximum depth of each tree included 10, 20, 40, 50, 60, 70, 80, 90, and 100. We performed a 5-fold cross validation for each combination and recorded each iteration’s performance using root mean squared error (RMSE). The parameter values giving the lowest RMSE was used to specify the final model. We applied this final model in the next steps, and we reported this model’s goodness-of-fit by applying it to the testing set as our first validation.

Using the gridded covariates as the input, we applied the final model to estimate a population weight for each grid cell. Next, we distributed township-level population to each grid cell using the population weight. Population weight in grid cells outside the inhabited zones was set to zero to avoid any population being distributed. Through this distribution, the sum of gridded population in a township should equal its total population.

We iterated this dasymetric mapping process above for total population and population by age groups (0–14, 15–59, ≥60, and ≥65 years old) to obtain their respective gridded population estimates. For each grid cell, we compared the estimated total population with the sum of population by age groups. Specifically, we calculated the correlation and RMSE between the two sets of estimates to reveal their consistency with each other (i.e., our second validation).

## Data Records

ASPECT is deposited as GEOTIFF files with WGS 1984 geographic coordinate system (EPSG: 4326), at the Figshare repository (10.6084/m9.figshare.27323106.v1)^20^. The dataset includes GEOTIFF files on population density (persons per hectare) at 100 m spatial resolution. No data areas indicate townships missing the 2020 Census data (n = 590) and areas fall outside our study area, mainland China. Notably, for total population, two files are provided. The first file (“population_total_pop.tif”) is gridded total population estimates directly from dasymetric mapping. The second file (“population_total_pop_sum.tif”) is the sum of gridded population by age groups, which are estimated from their respective dasymetric mapping. As discussed later, total population estimates from the two files are in general consistent with each other.

**Fig. 4:**
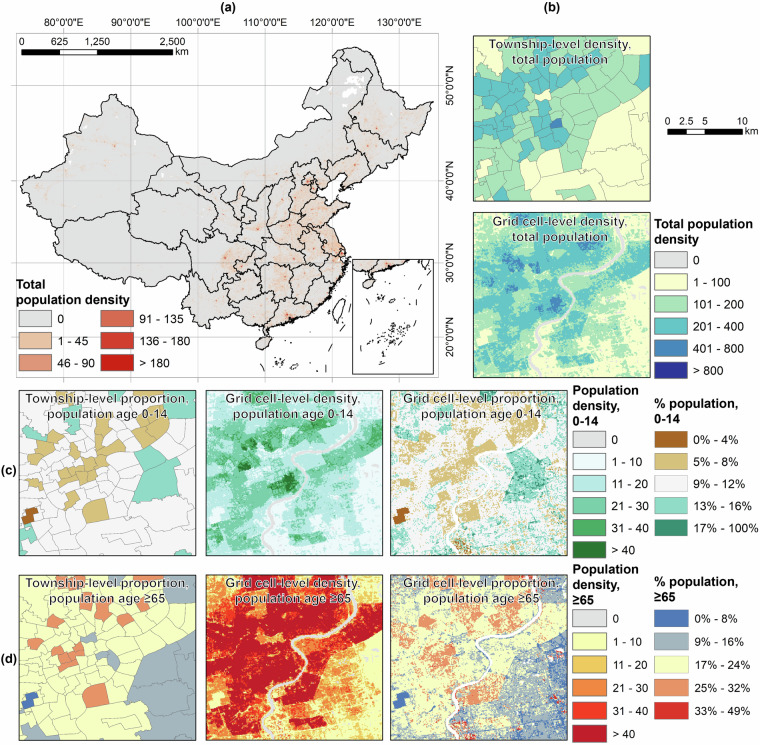
Illustration of ASEPCT: (**a**) total population density (persons/hectare) nationwide and (**b**) zoom-in. Density and proportion of population (**c**) 0–14 years old and (**d**) ≥65 years old are also illustrated.

ASPECT also includes GEOTIFF files on the proportion of population by age groups (0–14, 15–59, ≥60, and ≥65 years old) at 100 m spatial resolution. No data areas indicate townships missing the 2020 Census data, places fell outside our study area, and places with zero population. Proportion of a population age group is calculated by dividing its population counts by the sum of population from all age groups (i.e., grid cell values from the “population_total_pop_sum.zip” file). An illustration of ASPECT is provided in Fig. 4.

## Technical Validation

We performed three sets of validations on ASPECT. First, we evaluated the goodness-of-fit of the model regressing population density on the covariates at the township level. Second, for each grid cell, we compared the estimated total population with the sum of estimated age-group-specific population. Lastly, we performed the dasymetric mapping using county-level data, which is the next administrative level coarser than townships. We aggregated gridded population estimates from this county-level mapping by townships and compared with actual township-level values. The comparison helps to justify the validity of the dasymetric mapping, with the assumption that the final township-level mapping will have higher accuracy than this county-level mapping used for validation.

### Model goodness-of-fit

The random forest model fitting population density on covariates of % built-up area, building height, nighttime light intensity, distance to road, POI density, and topography at the township level achieved satisfactory accuracy (Table 2). The R^2^ was between 0.75 and 0.83, with the model predicting total population having the highest R^2^ and the model predicting population 0–14 years old having the lowest R^2^.

**Table 2:** Goodness-of-fit of model regressing population density on the covariates at township level.

Population group	R^2^	RMSE (persons/ha)
Total population	0.83	18.01
Age 0–14	0.75	3.36
Age 15–59	0.83	12.54
Age 60–64	0.77	1.32
Age 65 and above	0.78	3.19

**Fig. 5:**
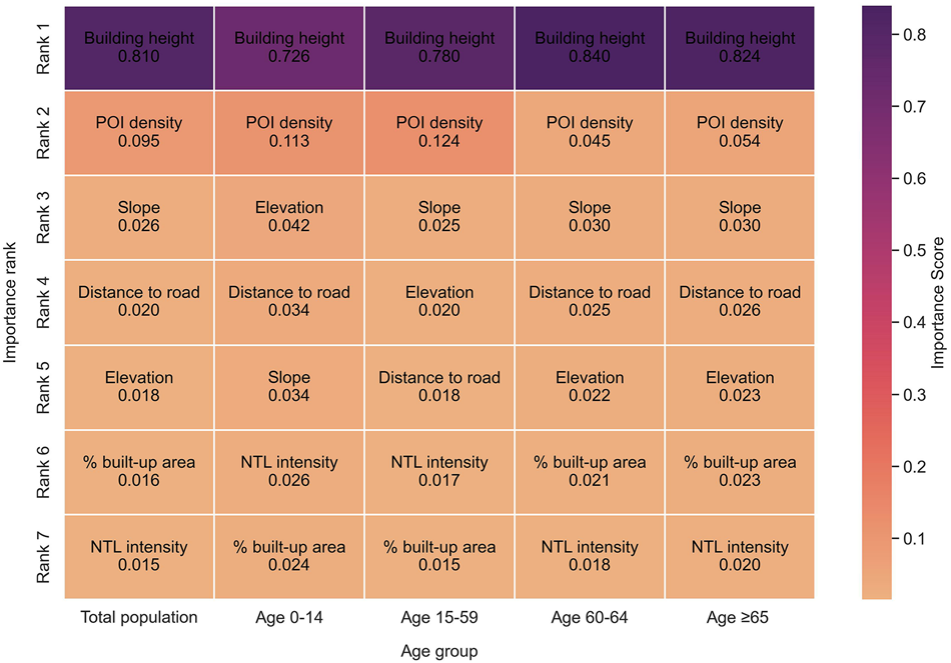
Covariate importance regarding different population groups.

**Fig. 6:**
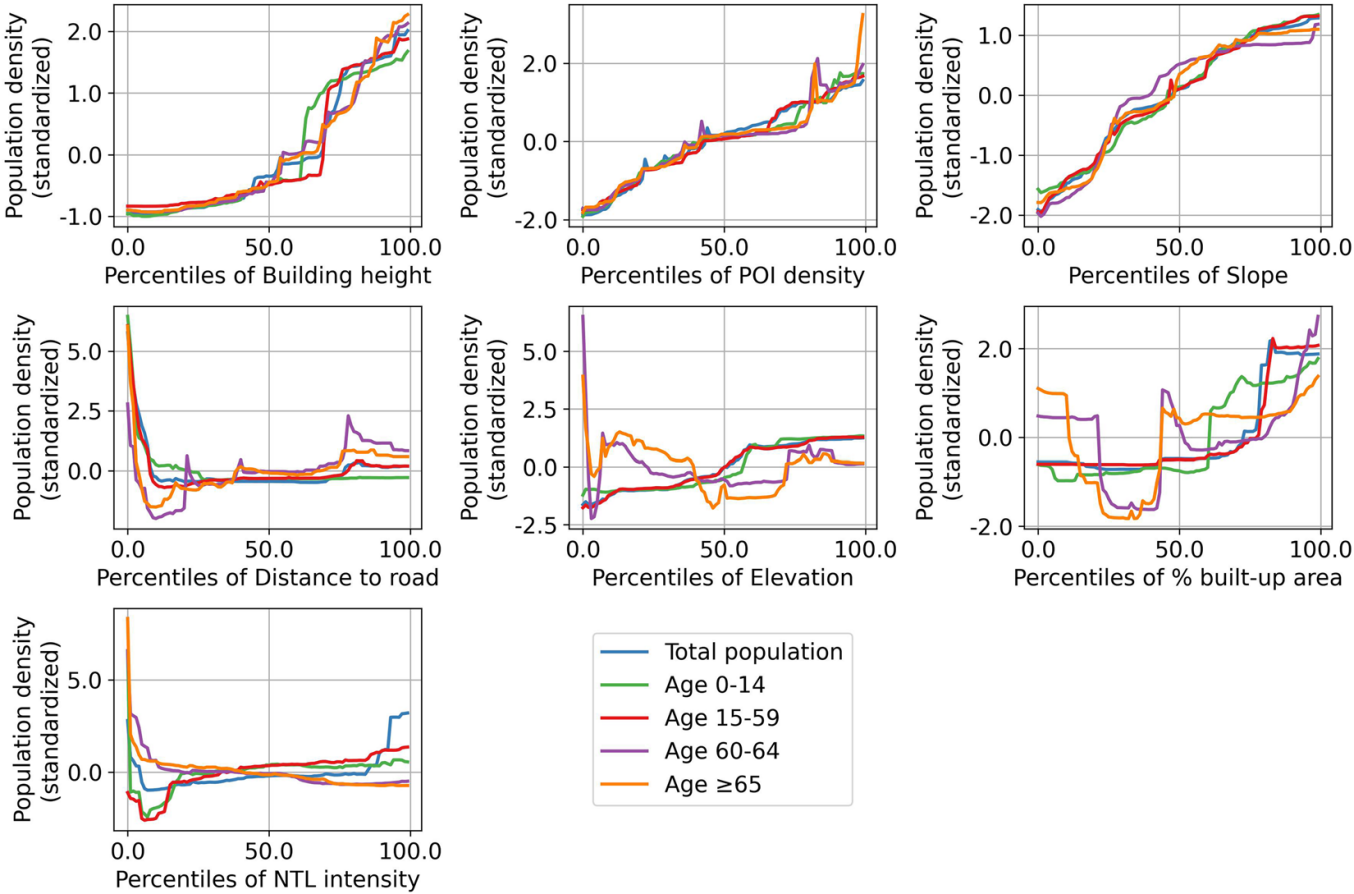
Partial dependence plot among the covariates and different population groups.

While using the same set of covariates for the dasymetric mapping, the importance of individual covariates in the random forest model varied across population groups (Fig. 5). Specifically, building height emerged as the strongest predictor for township-level population density, though its importance magnitude varied between population groups. POI density, slope elevation, distance to road, % built-up area, and NTL intensity remained as secondary predictors, with importance values substantially lower than building height. Notably, covariate importance rankings differed between population groups. While total population, population 60–64 years old and ≥65 years old shared identical rankings, they differed from those of population 0–14 years old and 15–59 years old. The differential effects of covariates on population density across population groups are further illustrated through partial dependence plots (Fig. 6), which estimate expected population densities corresponding to different covariate values.

### Comparison between gridded total population and sum of population by age groups

Our evaluation revealed that, in a given city (n = 366), the correlation between gridded total population and sum of age group-specific populations was averaged at 0.96 (interquartile range, IQR: [0.94, 0.98]). The average RMSE was at 5.47 (IQR: [4.09, 6.33]) persons/hectare. These results indicated that, despite some limited differences, the gridded total population was generally consistent with the sum of gridded population by the age groups. Therefore, we used the sum of population by age groups as the denominator to calculate population age structure per grid cell.

### Dasymetric mapping with county-level data

The ideal dataset to validate APSECT is gridded actual population counts at 100 m resolution, which is however not available. Here, we followed a validation approach employed by the previous literature^8^, which uses a spatial unit that is coarser than the finest one available to estimate gridded population (i.e., county instead of township). The resulting gridded population is then summed by the finest spatial unit available (i.e., township) and compared with the actual population in this unit. The underlying assumption is that the accuracy of dasymetric mapping from the coarser spatial unit is likely lower than that from the finer spatial unit; thus, this assessment with county-level data provides a lower bound for the accuracy of the final mapping with township-level data.

**Fig. 7:**
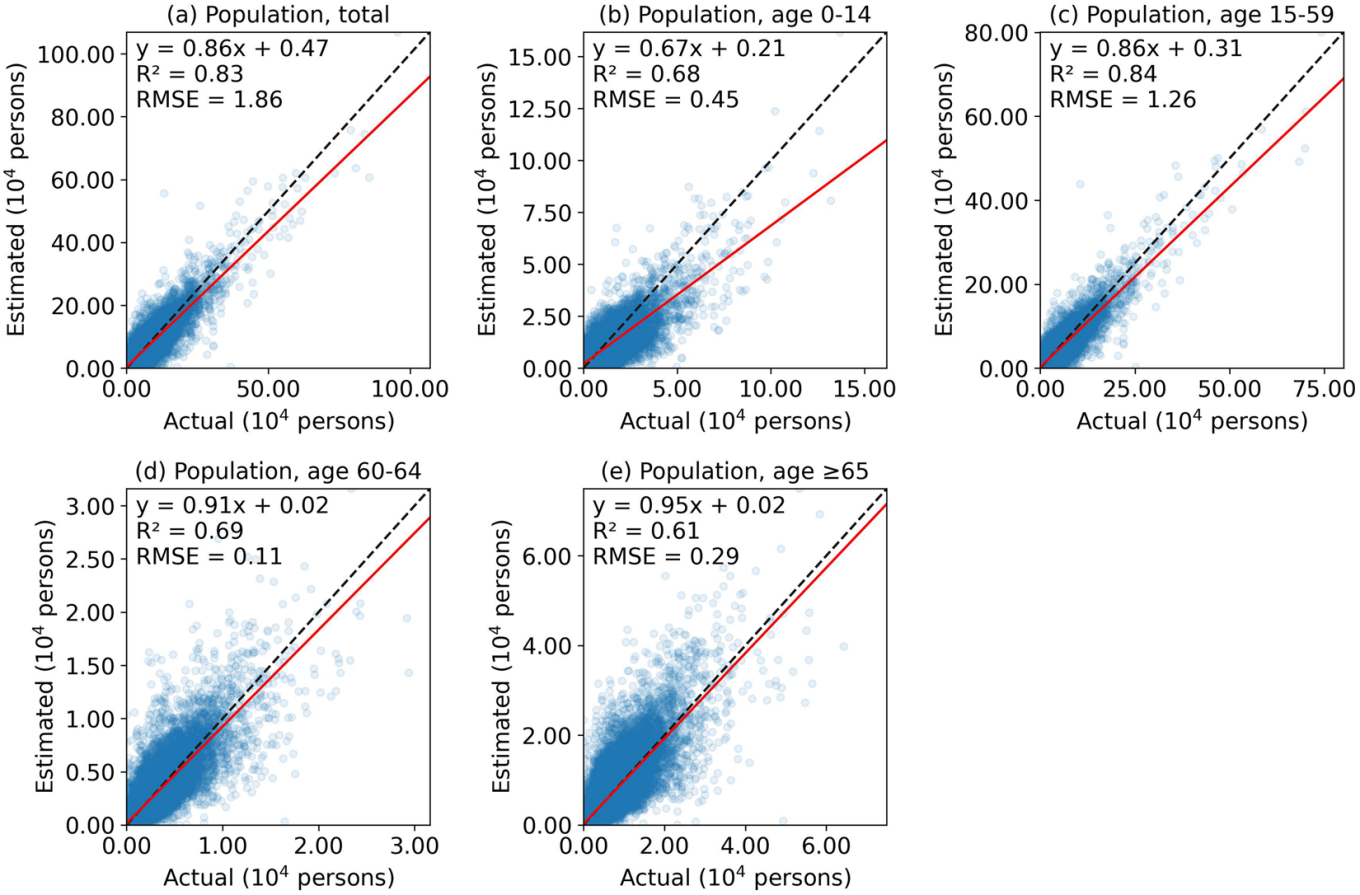
Accuracy of ASPECT produced from county-level data. Here, ASPECT from county-level data is summarized by townships to estimate total population and population by age groups. These estimates are then compared against observed township-level populations. Red line: fitted, dashed line: identity line.

We performed this validation using county-level population data and the same dasymetric mapping covariates and process described above. We summed the resulting gridded population estimates by townships and compared the results with the actual township-level population. The results indicated that the estimated township-level population had good agreement with the actual population, with R^2^ between 0.61 and 0.84, and RMSE between 1,000 and 19,000 persons, pending on the population group (Fig. 7). All the regression coefficients were below 1, indicating that the dasymetric mapping tends to underestimate in more populated areas, particularly for population between 0–14 years old (Fig. 7b). When stratifying cities by population size, more populous cities exhibited stronger agreement between estimated and actual township-level values for total population and populations aged 0–14 and 15–59 years old, according to R^2^ (Table 3). However, township-level populations aged 60–64 years old and ≥65 years old were more accurately predicted in smaller cities. Since ASPECT is produced with township-level data, its accuracy is likely higher than this gridded dataset produced with county-level data.

**Table 3:** Accuracy of ASPECT produced from county-level data, stratified by city population size.

Accuracy metric	Population group	City by total population size				
		<0.5 million (n = 17)	0.5–1 million (n = 20)	1–5 million (n = 210)	5–10 million (n = 75)	>10 million (n = 19)
R^2^	Total population	0.72	0.71	0.76	0.83	0.85
	Age 0–14	0.62	0.63	0.59	0.68	0.75
	Age 15–59	0.70	0.71	0.77	0.84	0.86
	Age 60–64	0.79	0.74	0.68	0.67	0.61
	Age 65 & above	0.75	0.70	0.60	0.53	0.53
RMSE (10^4^ persons)	Total population	1.11	1.05	1.58	1.95	2.86
	Age 0–14	0.25	0.23	0.42	0.50	0.56
	Age 15–59	0.77	0.70	1.04	1.30	2.05
	Age 60–64	0.04	0.05	0.09	0.11	0.19
	Age 65 & above	0.11	0.13	0.24	0.32	0.45

Note: City size categories follow the State Council of China’s classification: small (<0.5 million), medium (0.5–1.0 million), large (1.0–5.0 million), very large (5.0–10.0 million), and extremely large (>10.0 million)^27^.

### Comparison with WorldPop

We additionally assessed the quality of WorldPop, a widely adopted population map also with age-group-specific estimates, to justify the advantage of ASPECT. Specifically, the year 2020 data from two WorldPop versions were used: the first is the 2000–2020 version (with country totals adjusted to United Nations population estimates), which is the most recent final release^21^ (https://hub.worldpop.org/geodata/summary?id=50346); the second is the 2015–2030 version (release R2024B V1) currently undergoing updates^12^ (https://data.humdata.org/dataset/worldpop-age-and-sex-structures-2015-2030-chn). Two assessments were conducted. First, we assessed how well population estimates from WorldPop match with actual population from the 2020 Census at the township level. Second, we evaluated how WorldPop and ASPECT reflected spatial variations in the estimated population age structure (e.g., proportion of population ≥65 years old). In the second assessment, we calculated the proportion of a given age group in each grid cell. We then extracted all grid cells within a spatial unit and calculated the variations in the estimated proportions. We repeated this process for all age groups (0–14, 15–59, 60–64, ≥65 years old) and administrative levels of township, county, city, and province.

**Fig. 8:**
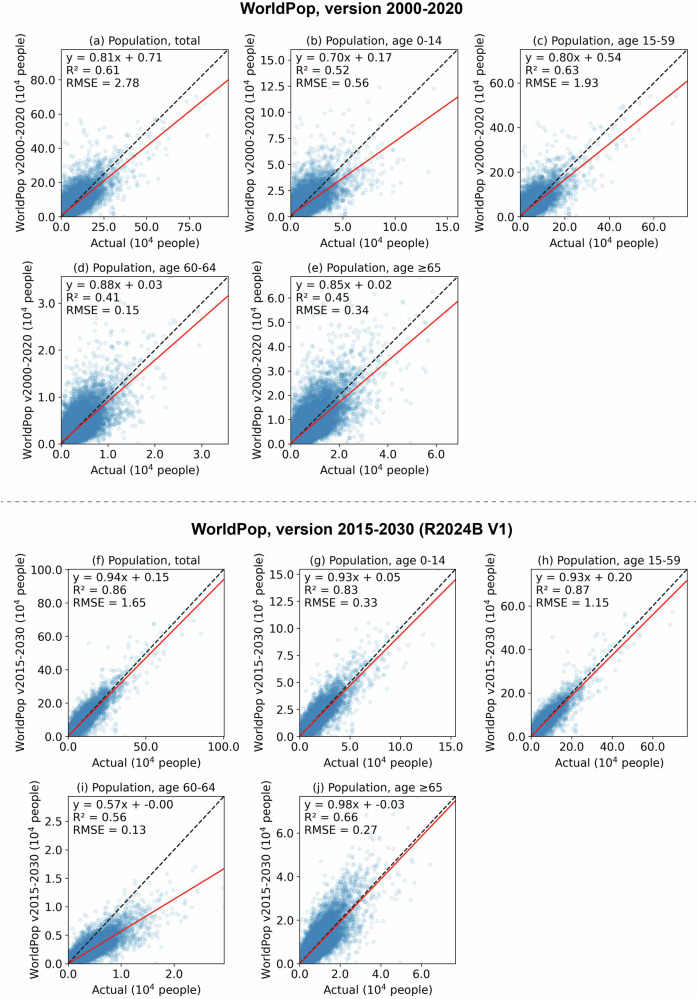
Accuracy of WorldPop against 2020 Census township-level data. Both WorldPop versions 2000–2020 (**a**–**e**) and 2015–2030 R2024B V1 (**f**–**j**) are assessed. Here, gridded WorldPop data for year 2020 is summed by townships to estimate total population and population by age groups, and they are compared with their respective estimates from the 2020 Census. Red line: fitted, dashed line: identity line.

Our first assessment indicated that, compared with ASPECT produced with county-level data (i.e., a lower bound on the prediction accuracy of the final ASPECT dataset, Fig. 7), WorldPop (version 2000–2020) likely exhibited larger errors when predicting township-level populations from the 2020 Census (Fig. 8a–e). Specifically, we found an R² between 0.41 and 0.63 and an RMSE between 1,500 and 28,000 persons when comparing WorldPop (version 2000–2020) with the 2020 Census at the township level (Fig. 8a–e). These agreements were lower than those from our estimates using ASPECT produced with county-level data (Fig. 7). The lower agreement and underestimation are likely due to the population projection methods used in WorldPop (version 2000–2020), as described in the Background & Summary section, which failed to accurately capture population changes between 2010 and 2020 at the township level. Meanwhile, WorldPop (version 2015–2030, release R2024B V1) showed improved performance in predicting township-level populations, with R² values between 0.56 and 0.86 and RMSEs between 1,300 and 16,500 persons (Fig. 7) – comparable to ASPECT produced with county-level data. Note that the county-level ASPECT likely represents a lower bound on the prediction accuracy of the final ASPECT dataset based on township-level data. Therefore, we assume that our final ASPECT dataset is at least comparable to, if not better than, WorldPop (version 2015–2030, release R2024B V1) in estimating gridded population counts, as the former is downscaled using township-level data, whereas the latter uses the coarser county-level data.

**Fig. 9:**
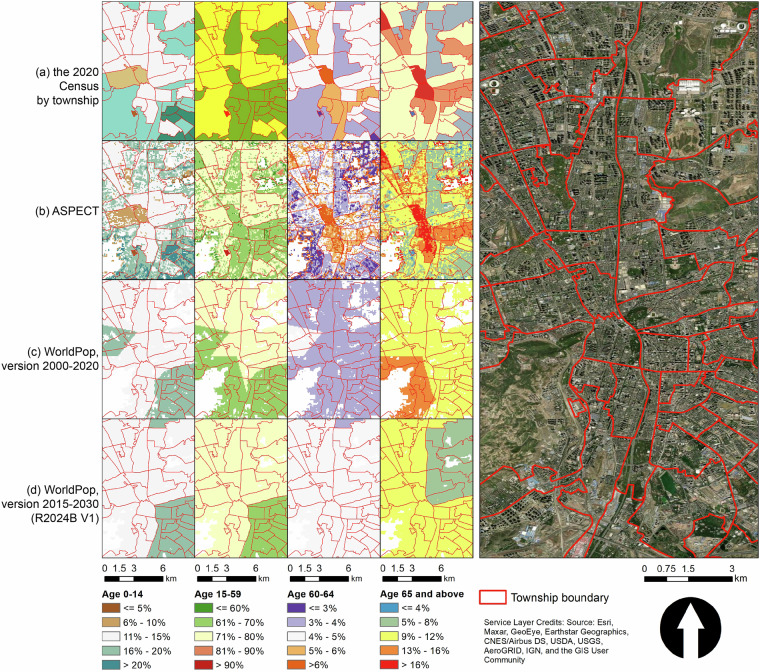
An illustration of the proportion of population by different age groups, estimated by the 2020 Census township-level data (**a**), ASPECT (**b**), WorldPop, version 2000–2020 (**c**), and WorldPop, version 2015–2030 R2024B V1 (**d**).

Our second assessment shows that, compared with ASPECT, both versions of WorldPop (2000–2020 and 2015–2030 R2024B V1) showed smaller spatial variations in the estimated proportion of population by age groups for year 2020. When summarized by townships, there were limited spatial variations in WorldPop’s age structure estimates (Table 4). The spatial variation in WorldPop’s age structure estimates increased when a larger spatial extent was used (i.e., province versus township). However, for a given spatial extent (i.e., province, city, county, or township), the spatial variations in WorldPop were between 2% and 30% of those in ASPECT. An illustration also revealed that ASPECT showed greater spatial variations in the estimated population age structure (Fig. 9b). This is because we iteratively conducted the dasymetric mapping to estimate population per age group for ASPECT. However, the estimates in WorldPop showed lower spatial variations and appeared to be truncated (Fig. 9c,d). It is likely that WorldPop multiplies county-level age structures by the gridded total population to estimate population in different age groups. Therefore, grid cells in the same county may share the same proportion of population from a specific age group.

**Table 4:** Spatial variations, measured by standard deviation, in gridded estimates on the proportion of population by age groups from ASPECT and WorldPop, summarized across different spatial extents.

Age group	Spatial extent	ASPECT mean [IQR]	WorldPop (ver. 2000–2020) mean [IQR]	WorldPop (ver. 2015–2030 R2024B V1) mean [IQR]
% 0–14	province	11.69 [10.41, 13.51]	3.47 [2.17, 4.64]	3.22 [2.08, 4.27]
	city	11.34 [9.44, 13.39]	1.99 [1.26, 2.43]	1.88 [1.16, 2.38]
	county	10.40 [8.19, 12.75]	0.51 [0.16, 0.61]	0.18 [0.06, 0.20]
	township	9.55 [6.54, 12.84]	0.22 [0.00, 0.23]	0.08 [0.00, 0.09]
% 15–59	province	13.23 [12.32, 14.12]	3.90 [2.78, 4.73]	4.03 [3.45, 4.48]
	city	12.35 [11.38, 13.25]	2.38 [1.52, 3.13]	2.57 [1.87, 3.18]
	county	11.86 [10.87, 12.94]	0.65 [0.19, 0.78]	0.27 [0.08, 0.30]
	township	10.33 [9.30, 12.07]	0.27 [0.00, 0.28]	0.12 [0.00, 0.13]
% 60–64	province	3.78 [3.29, 4.28]	0.79 [0.58, 0.90]	1.00 [0.77, 1.21]
	city	3.58 [2.95, 4.23]	0.48 [0.32, 0.60]	0.62 [0.40, 0.76]
	county	3.56 [2.83, 4.22]	0.14 [0.04, 0.17]	0.07 [0.02, 0.08]
	township	3.30 [2.43, 4.11]	0.06 [0.00, 0.06]	0.03 [0.00, 0.03]
% ≥65	province	8.69 [7.67, 9.91]	2.07 [1.50, 2.34]	2.55 [2.11, 2.66]
	city	8.05 [6.91, 9.28]	1.23 [0.80, 1.55]	1.61 [1.04, 1.98]
	county	7.79 [6.53, 9.10]	0.37 [0.10, 0.44]	0.18 [0.05, 0.20]
	township	6.79 [5.18, 8.51]	0.15 [0.00, 0.15]	0.08 [0.00, 0.08]

### Comparison with PopSE by Chen *et al*

We also compared ASPECT with PopSE by Chen *et al*., which uses a hybrid of township-level and county-level data from the 2020 Census to estimate gridded population distribution^15^. Specifically, a sample of 15,564 townships and all counties are used to perform the dasymetric mapping, representing, to our best knowledge, the first effort to employ a large sample of 2020 Census township-level data. Compare with PopSE which estimates total population at the grid-level, ASPECT further estimates the spatial distribution of population by four age groups (0–14, 15–59, 60–64, ≥65 years old). Moreover, ASPECT uses a larger number of townships for the dasymetric mapping (n = 40,718, Fig. 1). This refined source data may better capture spatial variations in population distribution, at least between the townships.

## Usage Notes

The files of ASPECT, which are in GEOTIFF format, can be processed by GIS software such as ArcGIS and QGIS, and by programing language packages such as Rasterio in Python.

## Data Availability

The Python code for generating ASPECT is available on GitHub (https://github.com/yangju-90/ASPECT). In the repository, we also provided an external link to a sample of the required input data for producing ASPECT.
